# A Wolf in Sheep's Clothing: SV40 Co-opts Host Genome Maintenance Proteins to Replicate Viral DNA

**DOI:** 10.1371/journal.ppat.1002994

**Published:** 2012-11-08

**Authors:** Gregory A. Sowd, Ellen Fanning

**Affiliations:** Department of Biological Sciences, Vanderbilt University, Nashville, Tennessee, United States of America; University of Florida, United States of America

Simian virus 40 (SV40) was discovered in 1960 as a contaminant in early polio vaccines. Its discovery coincided with an explosion of knowledge in the new field of molecular biology, and SV40 was quickly adopted as a model to study eukaryotic genome structure, expression, replication, and cell growth regulation in cultured cells [1]. With a genome of only 5.2 kbp, SV40 relies heavily on host cell machinery to propagate, affording investigators a powerful tool to discover key host proteins that the virus manipulates. Indeed, a single multifunctional viral protein, the large tumor (T) antigen (Tag) (Figure 1A), is sufficient to orchestrate the replication of the viral mini-chromosome in infected monkey cells [2], [3]. The origin DNA binding domain of Tag binds specifically to the viral origin of DNA replication, and the C-terminal helicase domain of Tag unwinds parental DNA at SV40 replication forks. The development of a cell-free reaction containing purified Tag and primate cell extract enabled the identification of ten evolutionarily conserved host proteins that are necessary and sufficient, together with Tag, to replicate SV40 DNA in vitro [3], [4].

## Initiation: How Does Tag Recognize Origin DNA?

**Figure 1:**
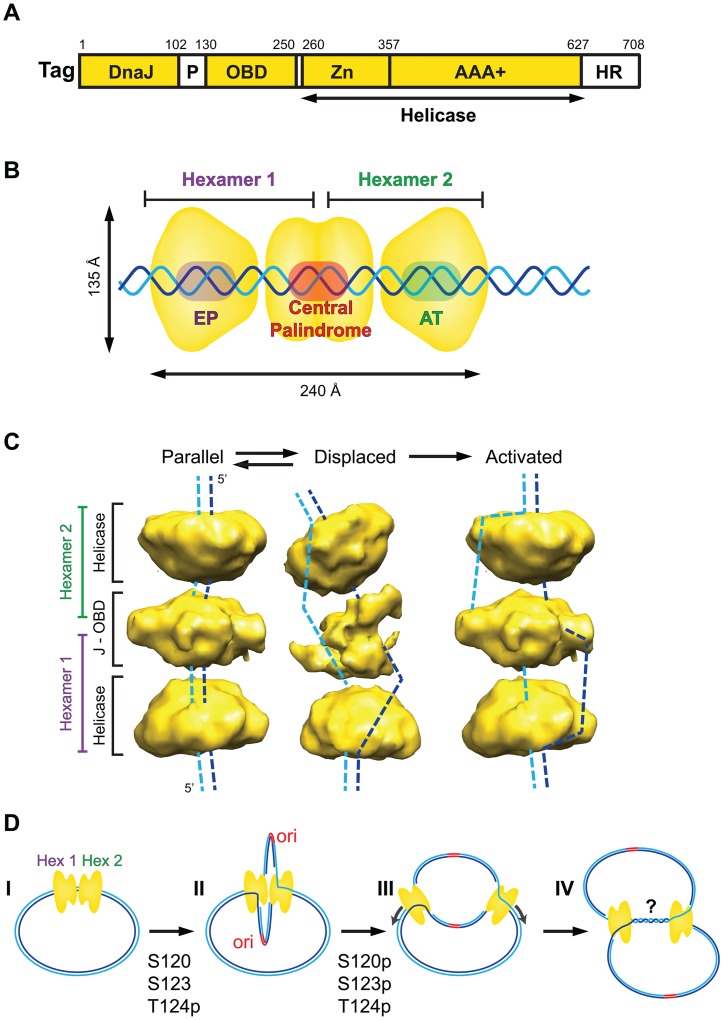
Assembly and activation of the SV40 pre-replication complex in vitro. (**A**) Domain architecture of SV40 Tag. Three structured domains (yellow) (DnaJ chaperone domain, origin DNA binding domain [OBD], and helicase domain), composed of the zinc (Zn) and AAA+ ATPase sub-domains, are connected by flexible regions (white) (P, cluster of phosphorylated residues that regulates origin activation; HR, host range function). (**B**) Diagram of ADP-associated SV40 Tag double hexamer bound to the duplex SV40 core origin of DNA replication (EP, central palindrome, AT), with non-origin DNA protruding from the complex (adapted from [6]). (**C**) 3D cryo-electron microscopy reveals two conformations (parallel, displaced) of ADP-associated hypo-phosphorylated SV40 Tag double hexamer on SV40 origin DNA as in (B) (adapted from [6]). A hypothetical conformation for the activated double hexamer is shown at the right. Dashed lines suggest potential paths of the DNA strands through each protein conformation. (**D**) Stages of SV40 replication. I, Tag dodecamer assembled on duplex SV40 DNA as in (B); II, hypo-phosphorylated Tag dodecamer activated as in (C) unwinds DNA bidirectionally [13] and may assemble host proteins (not shown here) into two sister replisomes that interact physically through the central lobe of the Tag dodecamer; III, hyper-phosphorylation of Tag disrupts interactions between the hexamers [7], [8], releasing the replisomes to progress independently along the template chromatin; IV, replication forks converge slowly, accompanied by DNA decatenation, to complete replication, which may involve additional host proteins [3], [14], [18]–[21].

Assembly of Tag on the viral core origin of DNA replication (64 bp) is the first step in replication [3], [5]. The core origin DNA is composed of three elements: a central palindrome composed of four GAGGC sequences, flanked by a so-called EP element and an asymmetric AT-rich element (Figure 1B). Binding of a Tag monomer to each GAGGC in the central palindrome nucleates cooperative assembly of additional Tag to form a double hexamer of ∼1 MDa (Figure 1B). The central lobe of the dodecamer consists of the N-terminal 250 residues of both Tag hexamers ([6] and citations therein). The C-terminal helicase lobe of each hexamer (residues ∼260–708) interacts with the EP or AT element of the origin DNA. This pre-replication complex, in the presence of Mg-ADP or -ATP, is sufficient to locally melt (EP element) or untwist (AT element) duplex origin DNA. These local distortions are necessary, but *not* sufficient, to activate bidirectional helicase activity of the Tag complex in vitro or in vivo.

## Activation of Replication: How Does the Tag Double Hexamer Unwind DNA?

Activation of the double hexamer on origin DNA requires a unique phosphorylation state of Tag: phospho-Thr124, and unmodified Ser120 and 123 [7], [8]. Cooperative interactions between the N-terminal regions of the two hexamers during assembly on the origin require this same hypo-phosphorylated form of Tag, which, fortuitously, is expressed by recombinant baculovirus. When hypo-phosphorylated Tag double hexamers assemble in the presence of Mg-ADP, which prevents helicase activity, they adopt two distinct conformations [6] (Figure 1C). In one conformation (parallel), the duplex core origin DNA is buried in the central channel of the double hexamer. In each hexamer, the six origin DNA binding domain (OBDs) form a left-handed spiral structure surrounding the central palindrome [6], [9]. In the displaced conformation, the central lobe of the dodecamer is more open, yielding a bent structure. Intriguingly, bacterially expressed Tag double hexamer displays only the parallel conformation, consistent with its inability to activate bidirectional origin unwinding [3], [6], [7]. Thus, we suggest that conformational changes in the central lobe, in concert with local distortions in the EP and AT elements bound to the helicase lobes, may allow single-stranded DNA (ssDNA) release from the central channel of the double hexamer (Figure 1C, dashed lines). Hypothetically, the displaced protein conformation could shift back to the parallel conformation without fully recapturing both strands of ssDNA. Indeed, the observation that Tag double hexamer-ADP-origin DNA complexes dissociate into single hexamers after exposure to a single-strand-specific nuclease argues that ssDNA must become accessible outside of the protein complex [10]. The observed conformational flexibility [6] could thus generate an activated dodecamer poised for bidirectional unwinding by steric exclusion, as proposed for the cellular Mcm2-7 replicative helicase [11], [12]. Future studies to define the path of the DNA through an active Tag helicase complex will be required to test this model.

## Elongation and Termination: Is Movement of Sister Replication Forks Coupled?

Both unphosphorylated and phosphorylated forms of Tag assemble double hexamers on duplex SV40 origin DNA (Figure 1D, I). However, only the hypo-phosphorylated form of Tag displays cooperative interactions between the two hexamers and undergoes remodeling to activate the helicase to unwind with 3′ to 5′ polarity (Figure 1C, right). In vitro, purified hypo-phosphorylated Tag can unwind origin DNA bidirectionally without disrupting the cooperative interactions between the two hexamers, resulting in “rabbit-ear” DNA structures detectable by electron microscopy [13]. If this looped template were replicated, DNA synthesis at the two sister replisomes might be coupled (Figure 1D, II). However, in infected primate cells, most of the Tag is additionally phosphorylated on Ser120 and Ser123. Alanine substitution of either residue abolishes viral DNA replication in vivo [7], [14], implying that modification of both sites is important for replication. Since phosphorylation of Ser120 or Ser123 disrupts cooperative interactions between hexamers, we suggest that hyper-phosphorylation of Tag uncouples the two replisomes soon after initiation of replication (Figure 1D, III). Since hyper-phosphorylation of Tag has no detectable effect on its unidirectional helicase activity [3], [7], [8], the sister replication forks could migrate independently and converge to complete replication in vivo (Figure 1D, IV).

## SV40: A Simple Model for Host DNA Replication?

**Figure 2:**
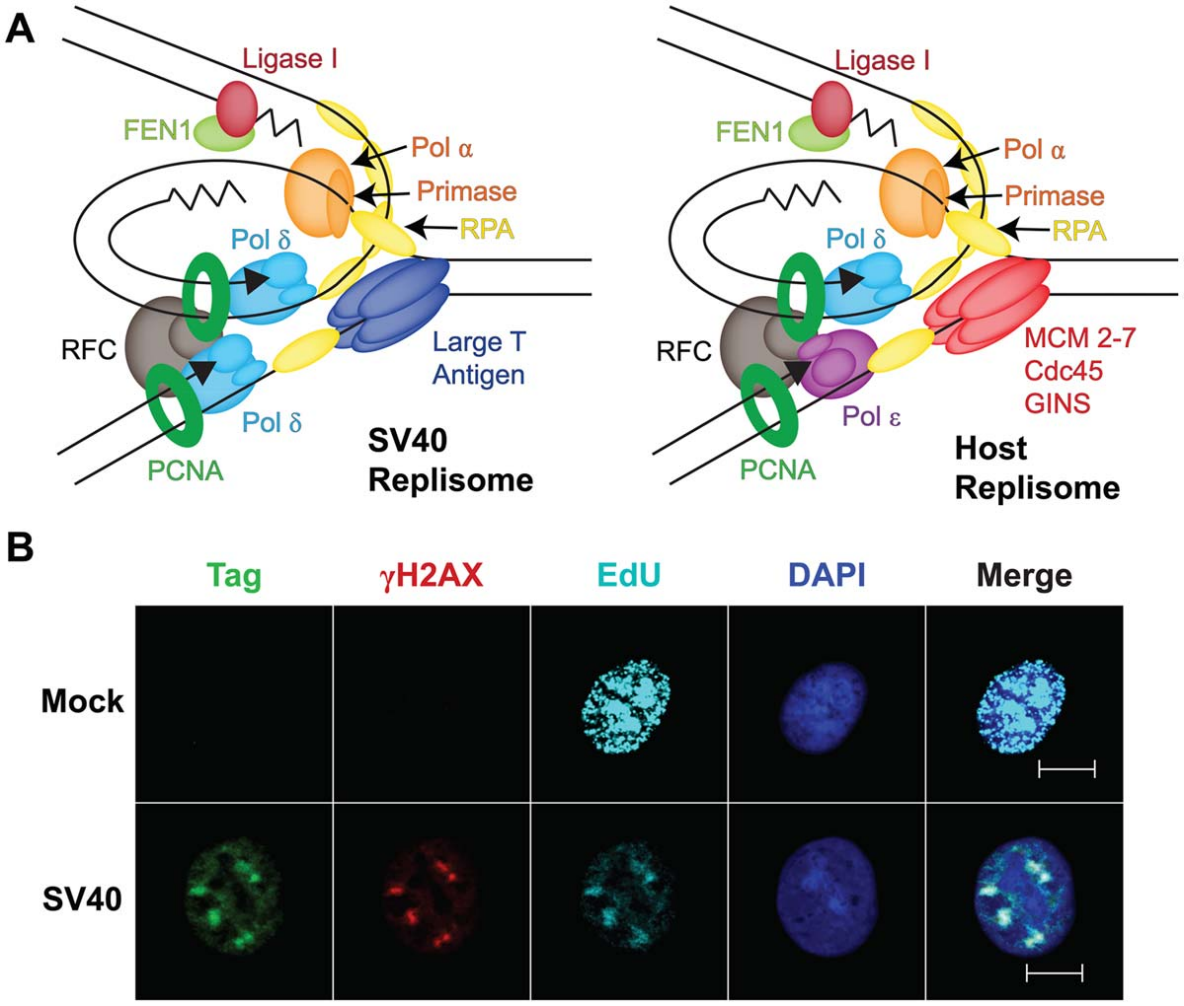
Viral exploitation of host DNA genome maintenance proteins. (**A**) Diagram of a minimal replication protein assembly (replisome) at a viral and a host fork. Topoisomerases, nucleosomes, and chromatin modifiers known to act at both forks are not shown (adapted from [24]). (**B**) DNA damage signaling in SV40 DNA replication centers at 48 hours post-infection, but not in host DNA replication centers. Mock-infected or SV40-infected BSC40 monkey cells were labeled with 10 µM EdU (a thymidine analog) for 5 minutes to visualize newly replicated DNA. Soluble proteins were pre-extracted and cells were fixed [18]. EdU (teal) was coupled to a fluorescent dye using click chemistry (Invitrogen) and DNA was stained with DAPI. Chromatin-bound Tag (green) and histone γH2AX (red) were stained for indirect immunofluorescence as described [18]. Cells were visualized with a 63× objective at a 0.6 µm z-axis slice using an Apotome (Zeiss). Scale bars represent 10 µm.

Investigation of SV40 replication has been motivated in part by anticipation that it would provide insight into host replication proteins and mechanisms. The architecture, dimensions, and assembly of Tag and yeast Mcm2-7 double hexamers on their cognate origin DNAs are closely related [6], [15], [16]. Much of the protein machinery at SV40 and host replication forks is also remarkably similar [2]–[4] (Figure 2A). Furthermore, the SV40 genome replicates in vivo as a mini-chromosome packaged in host nucleosomes and utilizes a variety of chromatin remodeling proteins and histone chaperones. Yet, the SV40 replisome clearly excludes several key components of host replication forks, e.g., the leading strand DNA polymerase ε, Mcm10, and Cdc45 ([17], [18]; G. Sowd, unpublished data), and all of the host proteins essential for SV40 replication in vitro (Figure 2A) function in host DNA repair, as well as replication, pathways. Lastly, SV40 infection induces host DNA damage signaling that is required to replicate viral chromatin in vivo [14], [18], [19]. These observations have prompted a re-evaluation of the viral replication strategy as a model for host chromosomal replication, and suggest the possibility that the virus may co-opt host repair pathways.

## Host Genome Maintenance: A Niche for Viral Chromatin Replication?

Recently, fluorescence microscopy of SV40 chromatin replication in infected cells has revealed that Tag and the host proteins required for SV40 replication in vitro co-localize in prominent subnuclear foci that enlarge with time after infection in permissive cells [18] (Figure 2B). Moreover, thymidine analogs, e.g., EdU, that are incorporated into nascent viral chromatin co-localize with these proteins, suggesting that these foci represent viral replication centers ([20], [21]; G. Sowd, unpublished data) (Figure 2B). Intriguingly, a variety of host DNA damage signaling and repair proteins, e.g., γH2AX, Mre11, Nbs1, Rad51, and FancD2, also reside in SV40 replication centers [18], [20], [21] (Figure 2B). Although punctate foci of such genome maintenance proteins are observed in chromatin of uninfected cells exposed to DNA damaging agents, such foci are generally much smaller than SV40 replication centers [18]. Of note, the association of host genome maintenance proteins with viral replication centers is not unique to SV40 or polyomaviral infections, but also occurs in cells infected by other DNA viruses, including adeno-, papilloma-, and herpesviruses [22], [23]. These findings suggest that host damage signaling and genome maintenance pathways serve important, though still poorly understood, roles in viral propagation, and raise questions about how viruses activate damage signaling. The localization of host genome maintenance proteins at SV40 replication centers suggests the possibility that viral chromatin may masquerade as “damage” to attract host proteins needed for replication (Figure 2A). A second possibility is that replicating viral chromatin may suffer actual DNA damage that host genome maintenance proteins could then repair. In either case, the activation of DNA damage checkpoints controlled by ATR and ATM signaling may arrest SV40-infected cells in a pseudo-S/G2 phase state that provides conditions favorable for viral DNA amplification [19], [21], [23]. Thus, much remains to be learned about how SV40 infection activates DNA damage signaling and uses it to facilitate viral propagation.
